# A Simple Visual Ethanol Biosensor Based on Alcohol Oxidase Immobilized onto Polyaniline Film for Halal Verification of Fermented Beverage Samples

**DOI:** 10.3390/s140202135

**Published:** 2014-01-27

**Authors:** Bambang Kuswandi, Titi Irmawati, Moch Amrun Hidayat, Jayus, Musa Ahmad

**Affiliations:** 1 Chemo and Biosensors Group, Faculty of Pharmacy, University of Jember, Jl. Kalimantan 37, Jember 68121, Indonesia; E-Mails: t.irmawati@yahoo.com (T.I.); amrun.farmasi@unej.ac.id (M.A.H.); jayus.ftp@unej.ac.id (J.); 2 Halal Science & Technology Research Group, Faculty of Science & Technology, USIM, Bandar Baru Nilai, Nilai 71800, Negeri Sembilan, Malaysia; E-Mail: andong@usim.edu.my

**Keywords:** halal, polyaniline, alcohol oxidase ethanol, biosensor, fermented beverage

## Abstract

A simple visual ethanol biosensor based on alcohol oxidase (AOX) immobilised onto polyaniline (PANI) film for halal verification of fermented beverage samples is described. This biosensor responds to ethanol via a colour change from green to blue, due to the enzymatic reaction of ethanol that produces acetaldehyde and hydrogen peroxide, when the latter oxidizes the PANI film. The procedure to obtain this biosensor consists of the immobilization of AOX onto PANI film by adsorption. For the immobilisation, an AOX solution is deposited on the PANI film and left at room temperature until dried (30 min). The biosensor was constructed as a dip stick for visual and simple use. The colour changes of the films have been scanned and analysed using image analysis software (*i.e.*, ImageJ) to study the characteristics of the biosensor's response toward ethanol. The biosensor has a linear response in an ethanol concentration range of 0.01%–0.8%, with a correlation coefficient (r) of 0.996. The limit detection of the biosensor was 0.001%, with reproducibility (RSD) of 1.6% and a life time up to seven weeks when stored at 4 °C. The biosensor provides accurate results for ethanol determination in fermented drinks and was in good agreement with the standard method (gas chromatography) results. Thus, the biosensor could be used as a simple visual method for ethanol determination in fermented beverage samples that can be useful for Muslim community for halal verification.

## 1. Introduction

Halal verification and authentication of food products are an issue of major concern and one of these issues is related to the as halal verification of the alcohol content in foods, particularly in fermented beverages. From an Islamic point of view, alcohol is a serious matter and totally prohibited in food products. As food products are part of our daily life, Islamic Laws give a special significance to this issue. In Islam, foods containing alcohol are *haram* (prohibited or unlawful) for Muslim consumption [1]. Ethanol is the main constituent found in alcoholic beverages and other products that undergo fermentation. Alcoholic drinks are totally prohibited in Islam, and even a small amount of the drink added into foods or drinks will render the products *haram* [2], but trace amounts of ethanol (naturally present as in fermented beverage) are allowed if the amount is insufficient to cause intoxication, usually less than 1% [3].

Hence, developing analytical methods for halal verification is very important, especially for the Muslim consumers to protect them from prohibited or *haram* products and also to ensure product safety and quality. Conventional methods, such as HPLC, GC-MS & FTIR have been used for food analysis, where the food samples have to be sent to laboratories to analyze for the presence of alcohol. The process takes days and is very tedious. In addition, such methods are time consuming, are subject to sources of errors and discrepancies between laboratories, and need skilled personnel for operation of those expensive instruments [4]. Therefore, the development of alternative methods for ethanol determination which simplify the analysis is needed. If one could easily detect the presence of alcohol within minutes this would be very useful to the Muslim community for enforcement in determining the safe consumption of food products in terms of their halalness.

In this context there is therefore a need to explore alternative methods of ethanol detection for halal verification using a tool that is accurate, simple, low-cost, rapid, reliable and consumer-friendly. A biosensor is an excellent candidate for this purpose. Biosensors are versatile analytical tools, offering an attractive alternative for ethanol detection [5]. The use of enzyme-based biosensors for the detection of ethanol in complex samples offers better specificity and therefore, a simpler sample treatment. Alcohol oxidase (AOX) [6,7], NAD^+^-dependent alcohol dehydrogenase (ADH) [8,9] and PQQ-dependent alcohol dehydrogenases [10,11] have all been used as bioselective elements in ethanol biosensors.

Alcohol oxidase-based biosensors have an advantage over alcohol dehydrogenase biosensors, due to the fact the latter need the cofactor to be added to the sample or to be immobilised on the sensor surface, while AOX-based biosensors are simpler because they use only molecular oxygen (O_2_) for co-factor regeneration. The enzyme requires O_2_ to oxidize the ethanol and the products formed are acetaldehyde and hydrogen peroxide. Since, AOX enzymatically converts all primary alcohols and formaldehyde [12], it suffers from a lack of selectivity to ethanol. However, this should not be a problem in the use of such a biosensor for analysis of ethanol in fermented beverage samples, since ethanol is present at much higher levels. The main problem of AOX-based biosensors is their limited stability. For this reason, several ways of stabilizing AOX in the dry state using a combination of polyelectrolytes and sugar derivatives have been studied [13,14].

In this work the development of a novel and simple visual ethanol biosensor based on AOX immobilized onto a polyaniline (PANI) film is reported. PANI is a polymer that changes conductivity and colour with changes in pH or redox reactions as a result of changes in the degree of protonation of the polymer backbone, making it useful as an optical or a visual sensor. PANI film itself acts both as a matrix support compatible with biomaterial (e.g., enzyme) and as the indicator, and can be easily be fabricated [15,16]. Furthermore, PANI has already been reported as a polymeric matrix in chemical sensors [17–19] and biosensors [20–24] developments. In the case of a PANI-based biosensor, most employ a class of enzymes known as oxido-reductases, mainly oxidases and dehydrogenases. In the case of oxidases, they are mainly based on peroxidase, glucose oxidase, or cholesterol oxidase [25]. A few of them used lipase [26], invertase [27] and polyphenol oxidase [28], and very few of them have used AOX.

Here, we used AOX as enzyme catalyst for ethanol detection, coupled with the optical properties of PANI as a visual sensor, so that it the presence of the alcohol could be detected by the naked eye due to a colour change from green to blue. For quantitative detection, the colour change of the films towards ethanol has been scanned and analysed using image analysis software (*i.e.*, ImageJ). Optimisation of experimental conditions has been carried out and the analytical parameters of the biosensor have been determined. The operational and storage stability of the biosensor were also evaluated.

## 2. Experimental

### 2.1. Reagents and Solutions

Aniline (AR-grade), alcohol oxidase (AOX) (A2404, EC1.1.3.13, 10–40 units/mg protein, from *Pichia pastoris*), ascorbic acid (A5960), gallic acid (G7384) and l-cysteine (W326305) were purchased from Sigma-Aldrich (Saint Louis, MO, USA). Absolute ethanol (>99.5%), methanol, orthophosphoric acid 85% and sodium hydroxide (pellets) were delivered by Merck (Nottingham, UK). All chemicals were of analytical reagent grade. The Milli-Q water used was obtained from a Millipore Direct-QTM 5 purification system. Stock solutions of ethanol was prepared daily in 0.1 M phosphate buffer of suitable pH and stored at 4 °C in refrigerator. The phosphate buffer 0.1 M solutions of pH values between 4 and 8 were used for pH studies. The pH was measured using a commercial glass electrode and a pH-meter (model 9318, Hanna Instruments, Woonsocket, RL, USA) calibrated at the pH values of 4.00, 7.00 and 9.00.

### 2.2. PANI Film Preparation

Aniline was purified by distilled under vacuum with vigorous stirring to prevent bumping. A PANI dispersion was prepared as a nanofibre using the methods described by Huang and Kaner [29]. The purified aniline (3.2 mmol or 0.3 g) was mixed with 1.0 M HCl acid solution (10 mL). Ammonium peroxydisulfate (0.8 mmol or 0.18 g) was mixed into another aliquot (10 mL) of the acid solution. The aniline-acid solution was added to the oxidant and the two solutions were rapidly mixed for 30 s and then allowed to react undisturbed overnight. The following day, the polyaniline was washed with water and centrifuged. After three washings, the supernatant liquor with a pH of 3.3 and was strongly green in colour, indicating the presence of PANI particles. Before casting, any remaining particles larger than 1 μm were removed by passing the dispersion through a 55-mm glass fiber filter (Whatman GFA, Kent, UK) attached to a vacuum source. The PANI dispersion was cast directly on a polystyrene substrate. Then the thin film of PANI on the polystyrene sheet were left overnight in the dark to dry after which individual 10 mm^2^ sections were cut. The ready film was then stored at 4 °C. The film thickness was determined by SEM images to be 0.7 μm. The film thickness was routinely determined for film samples to make sure that the thickness was always within in the same order of magnitude. The PANI film of similar thickness (0.7 μm) was selected and used for further experiments for good reproducibility of the PANI film fabrication.

### 2.3. Enzyme Immobilization

The procedure used is the same in all cases. The PANI film was conditioned at pH 7.0 by immersion in pH 7.0 0.1 M phosphate buffer, then afterwards, an AOX solution of appropriate concentration (10 μL) was deposited on the PANI film, and left to dry 30 min. The PANI film with immobilised AOX was then stored at 4 °C for further use.

### 2.4. Biosensor Construction

**Figure 1.**
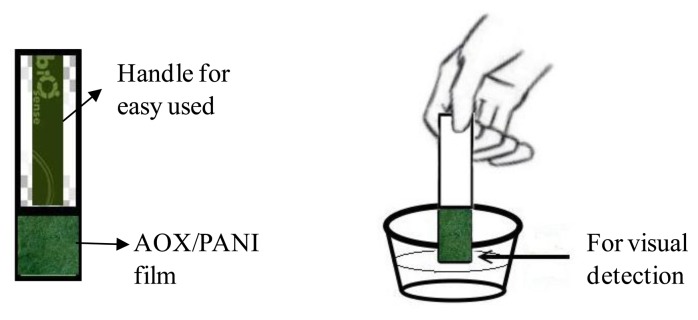
AOX/PANI film based biosensor design as a dip stick test (**a**) and simple used of sensor by dipped into sample solution (**b**).

The PANI film with immobilized AOX was constructed as a visual biosensor in the form of a dip stick test as shown in Figure 1, where the AOX/PANI film was connected to a cellulose paper as a handle for easy use using transparent plastic tape. In this dip-stick format, the biosensor could be easily used, just by dipping the biosensor into a sample solution for a few seconds (±5 s), then the color change of the biosensor could be detected visually using the naked eye for ethanol concentrations >1% (as this level is the allowable ethanol content in fermented beverages), and for quantitative measurement the color change could be further determined using color image analysis.

### 2.5. Colour Change Recording

Since the developed biosensor was intended to be used in visual mode, so that colour changes during alcohol detection can be easily viewed by the naked eye, for quantification of colour measurements of the indicator, a simple method using a scanner (Cano Scan, Canon, Tokyo, Japan) was used.

The colour change of the biosensor from green to blue was used as the measurable response of the biosensor toward ethanol, after the biosensor (as a dip stick test) was dipped to the sample solution for 5 s. The colour change of the biosensor was assessed using an ImageJ program, after it was scanned, to determine the mean RGB value of the colour. ImageJ is a public domain, Java-based image processing program developed at the National Institutes of Health [30]. It is also possible to solve many image processing and analysis problems using ImageJ's built-in editor and a Java compiler [31–34]. ImageJ can be run as an online applet, a downloadable application, or on any computer with Java 5 or a virtual machine [35,36]. The source code for ImageJ is freely available and has made image analysis simple, practical and affordable.

### 2.6. Interferences Measurement

Methanol, gallic acid, cysteine, ascorbic acid and sodium sulphite (as preservative agent) were checked as potential interferences with the colour change of the biosensor response. For all of them, an adequate dilution (1:10) in phosphate buffer 0.1 M pH 7 was the only sample treatment needed. The biosensor in dip stick format was dipped into different solution samples and the colour changes were recorded using the experimental procedure described above. In addition, catalase (C3515 EC 1.11.1.6, 4,000 units/mg from *Aspergillus niger*, Sigma, ∼10 units of catalase in phosphate buffer) was add to the sample that contained 1% of ethanol.

For application of ethanol detection in coloured solutionss, several colour interference tests have performed, by testing red wine (12% v/v, for red colour), orange (for yellow colour), apple (for brown) and grape juices (purple/dark colour). For all the fruit juices, 1% of ethanol was added to the samples. For all of them, an adequate dilution (1:10) in phosphate buffer 0.1 M pH 7 was needed for sample treatment. The biosensor was dipped in each different colored sample and after the color change, the fluid surrounding the surface of the biosensor was flushed gently with water to remove any colour bound on the surface, then the colour change was visually detected and recorded using the experimental procedure given above.

### 2.7. Real Sample Measurements

The developed biosensor was used to analyze some different alcoholic beverages (beer, legen (a Javanese traditional fermented drink) and tape (fermented glutinous rice)). In all cases, a 1:10 dilution in phosphate buffer 0.1 M pH 7 was the only sample treatment needed. Then, the colour change was recorded upon dipping the biosensor into 1 mL of each sample solution.

### 2.8. Gas Chromatographic Measurements

For comparison purposes, real samples were also analyzed by gas chromatography with a HP6890 chromatograph composed of an injector, a 2 m long packed column and a flame ionization detector (FID). The alcoholic beverages were analyzed using an internal standard method. A calibration plot for ethanol in concentrations ranging between 0 and 10% (v/v) was constructed using 5% of propanol as internal standard. The samples were previously diluted to obtain an adequate ethanol concentration.

## 3. Results and Discussion

### 3.1. Biosensor Scheme

The biosensor uses AOX (alcohol oxidase) as a bioselective element. AOX oxidizes low molecular weight alcohols to the corresponding aldehyde, using molecular oxygen (O_2_) as the electron acceptor, according to the following reaction:

(1) $${\text{RCH}}_{2}{\text{OH}+\text{O}}_{2}\overset{\text{AOX}}{⟶}{\text{RCHO}+\text{H}}_{2}{\text{O}}_{2}$$

**Figure 2.**
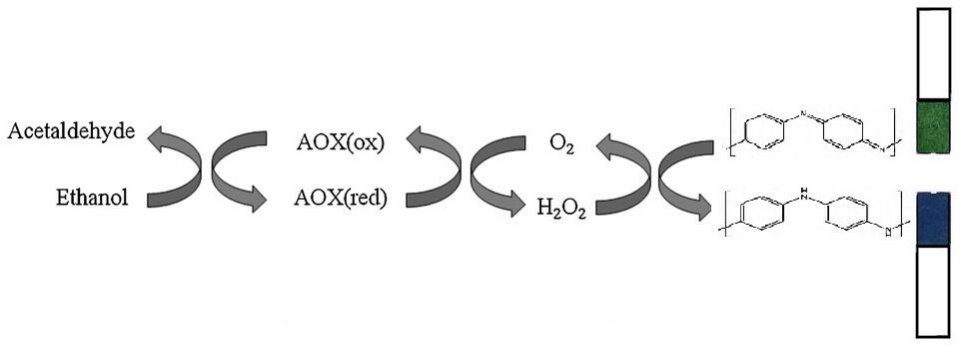
Proposed reaction mechanism at AOX/PANI film based biosensor towards ethanol.

The oxidation of alcohol by AOX is irreversible due to the strong oxidizing character of O_2_ and can be monitored by measuring either the decrease in O_2_ concentration or the increase in H_2_O_2_ concentration [37]. Here, the optical or visual determination of ethanol is based on the oxidation or reduction of H_2_O_2_ generated by the enzyme-catalyzed reaction. In order to shuttle electrons involved in the oxidation or reduction of H_2_O_2_ in optical or visual mode, the use of optical membrane or film is needed, such as polyaniline (PANI) film. Therefore, the enzymatic reaction could be monitored optically by the reduction of the PANI film from its emeraldine salt form (green) to the emeraldine base form (blue) as shown in Figure 2.

### 3.2. Optimisation of the AOX/PANI Biosensor

**Figure 3.**
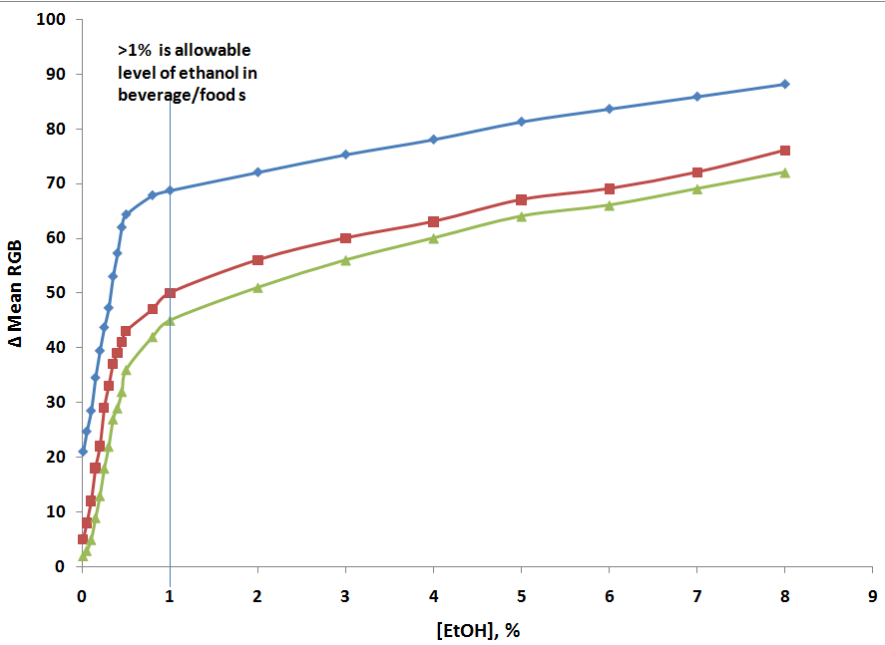
Calibration curves recorded at AOX/PANI biosensors using Δ mean RGB values in 0.1 M phosphate buffer at pH 6.0, with 0.01% (▲), 0.05% (■) and 0.1% (◆) AOX on PANI film.

The optimisation of variables affecting the AOX/PANI biosensor system was performed with respect to the amount of enzyme and with respect to pH buffer. In order to find the optimal amount of enzyme absorbed onto the PANI film, three different enzyme solutions were used to construct the biosensor, each containing AOX in concentrations of 0.01, 0.05 and 0.1%. These biosensors were then used for the visual determination of ethanol under the previously optimised conditions. Calibration curves are presented in Figure 3 and analytical data obtained from analysis of the curves are given in Table 1. Here, the biosensor has a linear response in the range from 0.1%–0.8% (*i.e.*, the intensity of green colour increases) with a correlation coefficient (r) of 0.996. The limit of detection of biosensor was 0.001%, afterward, a linear response was found in the 1%–8% range (*i.e.*, the intensity of the blue colour increased) with a correlation coefficient (r) of 0.996. This type of response was also reported using electrochemical methods [38].

The sensitivity continuously increased with the increase in enzyme concentration when the enzyme concentration is increased from 0.01% to 0.1%. However, at the same time, the linear range becomes smaller with an increase in enzyme concentration and in particular the colour change at 1.0% of ethanol was low, so that is not clearly visible to the naked eye for detection. Since the biosensor constructed using 0.1% enzyme solution showed a much higher sensitivity, in particular intense colour changes at 1% of ethanol for easy naked eye inspection, this level it was chosen for performing further experiments.

**Figure 4.**
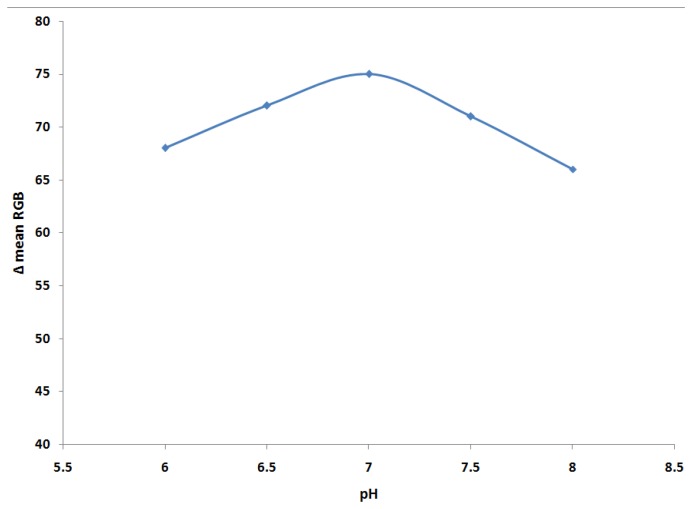
The effect of pH buffer on the AOX/PANI biosensor sensor toward ethanol (1%).

A study of the influence of the pH of the 0.1 M phosphate buffer on biosensor response was performed with the optimised AOX/PANI film as biosensor. The pH values of buffer solution were chosen in the range of 6.0–8.0, since it is known that the pH of AOX extracted from *Pichia pastoris* is 5.5–8.5 [39]. The results show an increase in the Δ mean of RGB values up to pH 7.0, above which the response decreases slightly (Figure 4). This also similar with previous studies that have shown that the highest enzyme activities and stabilities are achieved at physiological pH values [14,38].

### 3.3. Analytical Properties of the AOX/PANI Biosensor

#### 3.3.1. Response Time

The response time of the biosensor was counted (using a digital stopwatch with naked eye inspection), as a time needed to change the colour after the biosensor reacted with ethanol solution, just by dipping it into sample solution. The response time was found to be 5 s, which is a quite rapid response for this type of the biosensor. This might be due to the thin film (0.7 μm) used in this biosensor as well as the nanofibres of the PANI film, which make the color change really fast compared to other AOX based biosensors [20–24]. In addition, the response time of the biosensor was also recorded using a digital video camera (Canon PowerShot A4000) for quantifying the colour changes over time. It also found, in good agreement with the above procedure, that 5 s was 90% of the response time. Thus, for the response time, 5 s was used for further experiments.

#### 3.3.2. Reproducibility

**Figure 5.**
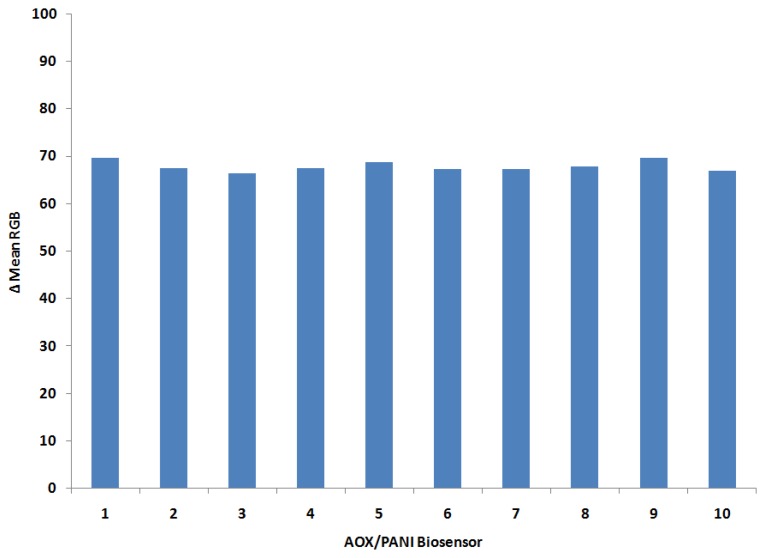
Reproducibility the AOX/PANI biosensor toward ethanol (1%) at pH buffer 7.0.

In order to examine the reproducibility of the biosensor, the biosensor response to ethanol was recorded under the same experimental conditions. The biosensors' response toward 1% of ethanol was tested as given in Figure 5, which shows good reproducibility (R.S.D. = 1.6%). This reproducibility is higher when compared with other reported AOX-based biosensors [20–24]. Since, the biosensor is intended for use as a one shot or disposable test, this study demonstrated a very small variation between produced biosensors in terms of response toward ethanol.

Most biosensor assemblies based on AOX consist of bi-enzyme designs where, together with the AOX, a peroxidase is used for hydrogen peroxide detection. Redox hydrogels are commonly used for this type of biosensor construction, but since this procedure implies manual mixing of both enzymes together with the cross-linker, problems may appear in the optimization procedure and reproducibility of these one-layer bi-enzyme biosensors [40,41]. The advantages of this newly developed biosensor assembly consist of its simple construction procedure and good reproducibility (0.6%). Another advantage of these new AOX/PANI biosensors is that they can be used for visual detection. Thus, the biosensor showed a sufficiently good reproducible behaviour and can be used for practical measurements of ethanol.

#### 3.3.3. Selectivity

Similar experiments were carried out for two other short chain aliphatic alcohols: methanol and 1-propanol and the analytical parameters obtained were compared. Table 2 summarises the analytical parameters calculated from the calibration curves recorded for each alcohol. As expected, on increasing the length of aliphatic alcohol chain, the sensitivity of the biosensor decreases as also shown in other studies [28,42].

#### 3.3.4. Stability

**Figure 6.**
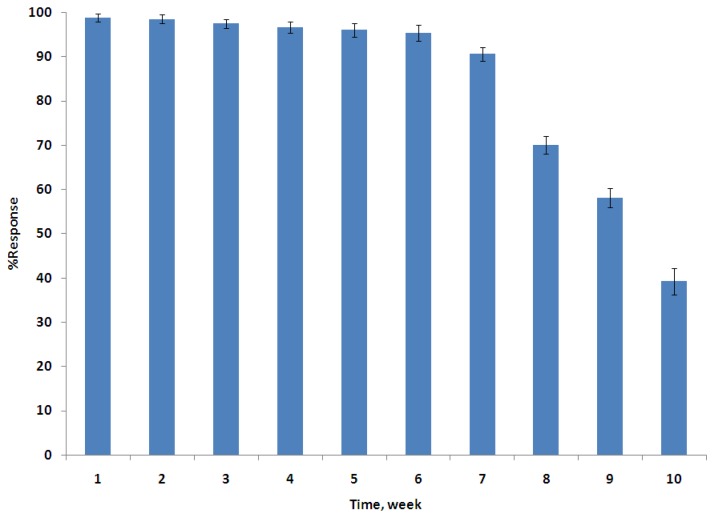
Stability of the AOX/PANI biosensor response (average of triplicate measurements) toward ethanol (1%) at pH buffer 7.0. within 1–10 weeks of storage (4 °C).

The AOX/PANI biosensor dip sticks were stored at 4 °C when not in use. In order to check the storage stability, a colour change of the biosensor toward ethanol (1%) was checked regularly every week. After 1 week, the biosensor response decreased by only 1.5%. A dramatic decrease was observed however after seven weeks of storage. The biosensor response decreased by about 9.5% from the initial value, which means that the biosensor could be used up to seven weeks, since within these periods the colour change of the biosensor can be detected by the naked eye. However, after eight weeks, the biosensor response continues to decrease, maintaining 70%, of the initial response as shown in Figure 6. This is very promising achievement with respect to the stability of the biosensor response, since it is well known from previous studies that AOX has a poor long-term stability which was its main disadvantage in previous biosensors [42–44].

### 3.4. Interferences

There are many easily oxidisable species present in beverage samples, the most important of which in the case of beer are ascorbic and tartaric acids. Since the purpose of this work was to use the biosensors as halal verification for ethanol content in fermented beverage samples, a study of interferences from compounds usually present in fermented beverages was needed. Several acids were examined as possible interferents that may affect ethanol determination in fermented beverage sample. The high level of selectivity towards common interferences provides the possibility to use this dip stick test in food, clinical, and environmental control. The results obtained are presented in Table 3. For the proposed AOX/PANI biosensor, quite small interference was found due to the oxidation of lactic acid and oxalic acid that can affect the PANI film as well as sodium sulphite used as preservative agent. The other acids are reduced, but also a small effect were found, even in the case of ascorbic and tartaric acids, in the presence of which the biosensor response to ethanol decreases by 1.3% and 4.7%, respectively, at a 2:1 ratio of interferent:ethanol.

In the case of the presence of catalase, this substance really gives a high interference with the biosensor response, since no distinct visual color change was observed at 1% of ethanol. This is due to the fact that the presence of catalse could reduce the biosensor response by ∼35% (using RGB values) and becomes a limitation of this biosensor. Since catalase is well known to decompose H_2_O_2_ under these experimental conditions (pH 7.0 and 25 °C), the presence of catalase will therefore compete with the PANI in the reaction towards H_2_O_2_, which in turn, reduces the biosensor response due the resulting H_2_O_2_ concentration decrease. However, in the case of fermented beverages catalase is expected to be absent or present in trace amounts [45], so in real sample conditions, the catalase should not affect much the biosensor response.

The results obtained for ethanol detection in coloured solutions are presented in Table 4. Here, a small interference was found at the AOX/PANI biosensor response due to coloured sample effects. This is due to the fact that some of coloured solution could bind to the surface of the biosensor. In order to remove this bound colour, it was necessary to flush the biosensor surface gently with water, so that the biosensor surface will remain clear and the response will be easy to detect by the naked eye or scanned for colour image analysis (ImageJ). In addition, the coloured solution was also reduced by a dilution (1:10) in phosphate buffer 0.1 M pH 7 as part of the sample treatment needed. Thus, the colored sample effect could be reduced to within the acceptable range of interferences (<5%).

### 3.5. Ethanol Analysis in Fermented Beverage Samples

The performance of the biosensor for practical applications in the analysis of fermented beverage samples was demonstrated by performing the determination of the alcohol content in beer, legen (a Javanese traditional fermented drink) and tapai (a solution obtained from fermented glutinous rice), estimated in terms of ethanol concentration. The samples just required a simple dilution step, in order to fit the linear range of the calibration curve. The alcohol content of the three types of fermented beverage samples was measured and summarized results are given in Table 5. The biosensor accuracy was assessed by comparison with the results given by the beer producers and others with the gas chromatography method.

## 4. Conclusions

Alcohol oxidase from *Pichia pastoris* has been immobilized by an absorption method onto PANI film as a visual biosensor in dip-stick format for determination of ethanol in fermented beverage samples. After optimisation of experimental parameters, the biosensor has a linear response in the ethanol concentration range of 0.0%–8.0% and a detection limit of 0.001%. The reproducibility is good (RSD of 1.6%) as well as the storage stability of the biosensor for up to 7 weeks at 4 °C. All these characteristics make the AOX/PANI biosensor a good alternative to other determination methods for ethanol as halal verification of the presence of alcohol in fermented beverage samples.

## Figures and Tables

**Table 1.** Analytical data obtained from the ethanol calibration curves of AOX/PANI biosensors.

**AOX (%)**	**Linear Range**	**Correlation** **Coefficient (r)**	**Limit Detection (%)**	**Visual Detection** **at 1% Ethanol**
0.01	0.01–0.6	0.985	0.01	Not easy
0.05	0.01–0.6	0.991	0.005	easy
0.1	0.001–0.8	0.996	0.001 (%)	Very easy

**Table 2.** Analytical data obtained from calibration curves of AOX/PANI biosensors (0.1% AOX) for three different alcohols under similar experimental conditions.

**AOX (%)**	**Sensitivity (Δ Mean** **RGB)/0.1%**	**Correlation** **Coefficient (r)**	**Limit Detection** **(%)**	**Visual Detection** **at 1% Ethanol**
Methanol	94	0.991	0.001	Very easy
Ethanol	91	0.996	0.001	Very easy
1-Propanol	63	0.985	0.005	Easy

**Table 3.** Interference of some compounds; % ratio 2:1 interference (2% v/v): ethanol (1% v/v), on the response to ethanol of AOX/PANI biosensors.

**Compound**	**Response of the Biosensor**
Ascorbic acid	98.7
Acetic acid	99.5
Lactic acid	102.9
Malic acid	96.5
Oxalic acid	101.6
Citric acid	95.8
Tartaric acid	95.3
Sodium sulfite	98.5

**Table 4.** Interference of coloured solution; in the presence of 1% (v/v) ethanol, on the response to ethanol of AOX/PANI biosensors.

**Compound**	**Response of the Biosensor**
Orange Juice	97.8
Grape Juice	103.6
Apple Juice	98.5
Red Wine *	105.3*

Note

* Recovered value.

**Table 5.** Determination of ethanol in the fermented beverage samples with AOX/PANI and by applying GC. Data are give as average ±SD (n = 3).

**Beverage Sample**	**Etanol (% v/v)**		

	**Value Declared by the Producer**	**GC**	**Biosensor**
Beer	4.9%	5.0 ± 0.5	4.27 ± 0.03
Legen	-	4.5 ± 0.7	4.31 ± 0.08
Tapai solution	-	1.4 ± 0.8	1.32 ± 0.02
